# Case report: a case of primary intracranial parasagittal meningeal angiosarcoma

**DOI:** 10.1186/s13000-023-01389-7

**Published:** 2023-09-16

**Authors:** Jun Cao, Jincheng Fang, Xiaochun Jiang, Guangfu Di, Jun Shen

**Affiliations:** Department of Neurosurgery, the First Affiliated Hospital of Wannan Medical College (Yijishan Hospital of Wannan Medical College), Wuhu, 241001 People’s Republic of China

**Keywords:** Meningeal tumor, Parasagittal angiosarcoma, Immunohistochemistry, Surgery, Treatment

## Abstract

**Background:**

Angiosarcoma, also known as malignant hemangioendothelioma, is a rare vasogenic malignant tumor, commonly found on the skin of the head and neck, rarely occurring in the intracranial region. As for intracranial meningeal angiosarcoma, only 8 cases have been reported before and there is no clinical study with large sample size. We report here a case of parasagittal meningeal angiosarcoma.

**Case description:**

A 48-year-old Chinese male patient was admitted to our hospital due to headache accompanied by bilateral lower limb weakness. On admission, CT showed a high-density mass on both sides of the sagittal sinus at the top of the frontal lobe. We performed exploratory surgical resection of the tumor. During the operation, it was found that the tumor originated from the dura mater and extensively invaded the surrounding brain tissue and skull, and the surrounding hemosiderin deposition was observed. Postoperative pathology suggested angiosarcoma.

**Conclusions:**

Intracranial meningeal angiosarcoma is difficult to accurately diagnose before surgery, so radiologists and neurosurgeons need to strengthen their understanding of this disease. The presence of extensive superficial hemosiderin deposition during operation may contribute to the diagnosis, and immunohistochemistry is very important for the diagnosis of intracranial angiosarcoma.

## Introduction

Angiosarcoma is a highly malignant tumor originating in vascular endothelial cells, accounting for approximately 1% of all sarcomas, can occur in any part of the body, most commonly in the head and neck, and usually has a poor prognosis [1]. Primary intracranial angiosarcoma is rare and may involve the brain or meninges [2]. At present, only a few cases of intracranial meningeal angiosarcoma have been reported, and no large sample studies have been conducted. To improve understanding of this rare disease, we present a case of primary parasagittal meningeal angiosarcoma in a 48-year-old man and describe its clinical course, imaging features, pathology, treatment, and prognosis.

## Case description

**Fig. 1:**
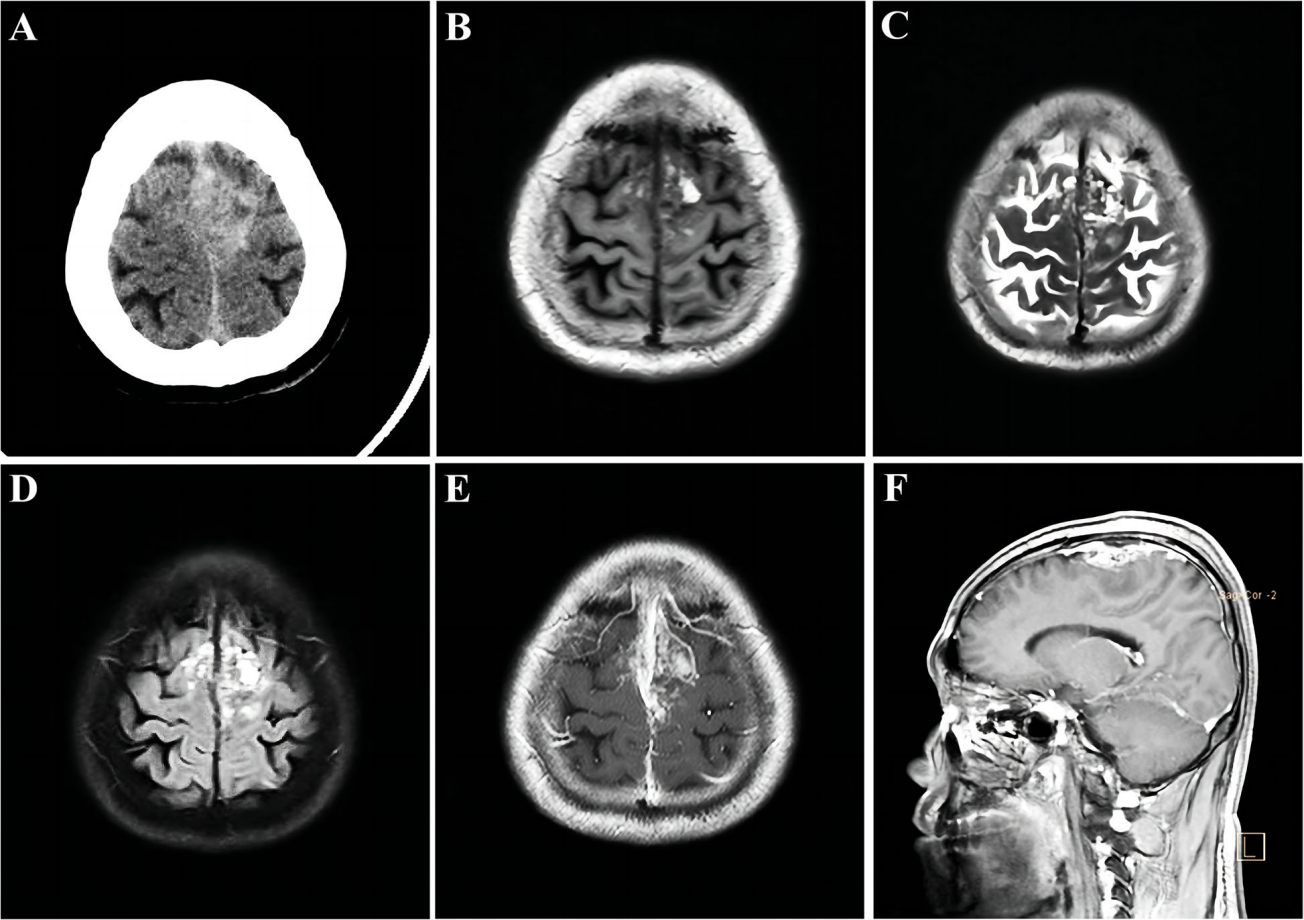
**A** Admission CT showed bilateral parasagittal high-density lesions, mainly on the left side, located in front of the motor area of the brain. **B** T1-weighted images and (**C**) T2-weighted image showed a parasagittal mass with irregular mixed signals, and some speckle high-signals was seen in it. **D** T2-FLAIR image showed mixed signals with ill-defined borders, encompassing the superior sagittal sinus, and invading the adjacent skull. **E** Axial T1-weighted images with contrast enhancement. **F** Sagittal T1-weighted image with contrast enhancement, showed uneven and obvious enhancement of the lesion, involving bilateral parasagittal dura and sagittal sinus, and local brain tissue compression

A 48-year-old Chinese male patient was admitted to our hospital with headache and bilateral lower limb weakness for 1 month. He had no previous history of specific diseases, no family history of diseases, and no history of radiation exposure. On admission, CT scan of the head revealed uneven high-density mass at bilateral frontal parietal lobe. The T1—and T2-weighted images of the head MRI showed mixed and uneven mass signals with insignificant surrounding edema. MRI enhancement showed uneven and obvious enhancement of the lesion, involving bilateral parasagittal dura and sagittal sinus, and local brain tissue compression (Fig. 1).

**Fig. 2:**
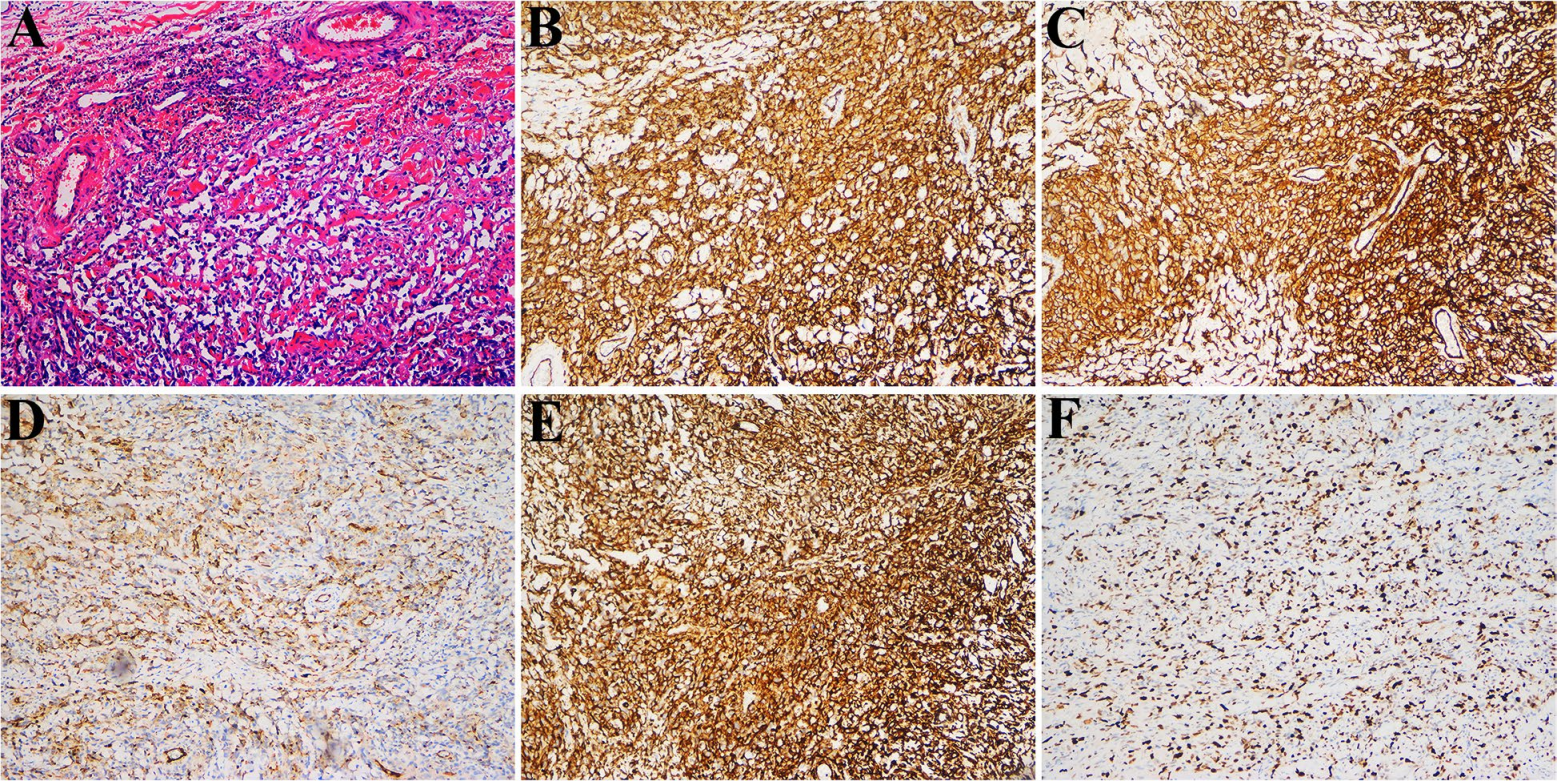
Histological and immunohistochemical features. **A** Hematoxylin and eosin staining of tissue samples showing the epithelioid appearance and angiogenesis of the tumor and a large number of new abnormal blood vessels, marked cell atypia, and marked nuclear staining. Immunohistochemical staining for vascular markers, such as CD31 (**B**), CD34 (**C**), factor VIII associated antigen (**D**), vimentin (**E**) and Ki-67 (**F**), showing strong positivity within the tumor cells. Pictures **A**, **B**, **C**, **D** and **E** were taken at 100 magnification, picture F was taken at 200 magnification

Subsequently, we performed bilateral tumor resection for the patient. During the operation, we found that the tumor originated from the dura mater. The tumor was found to be soft and reddish-brown, and the frontal and parietal cortex adjacent to the tumor was brownish yellow, which may be related to hemosiderin deposition. The tumor contained a large amount of brownish yellow fluid, and local skull and brain tissue were found to be invaded. Because the lesion was located in front of the motor center of the brain, the affected brain tissue could not be completely removed in order to preserve the motor function of the limbs. After surgery, the excised mass and the affected dural tissue were sent for pathological examination, and the final pathological result was angiosarcoma. Hematoxylin and eosin staining of tissue samples showed a large number of new abnormal blood vessels, marked cell atypia, and marked nuclear staining. The tumour cells were positive for the vascular markers such as CD31, CD34, and ERG, and negative for S-100, pancytokeratin, and epithelial membrane antigen. Ki-67 showed a hight index of positivity within the tumour cells (Fig. 2).

**Fig. 3:**
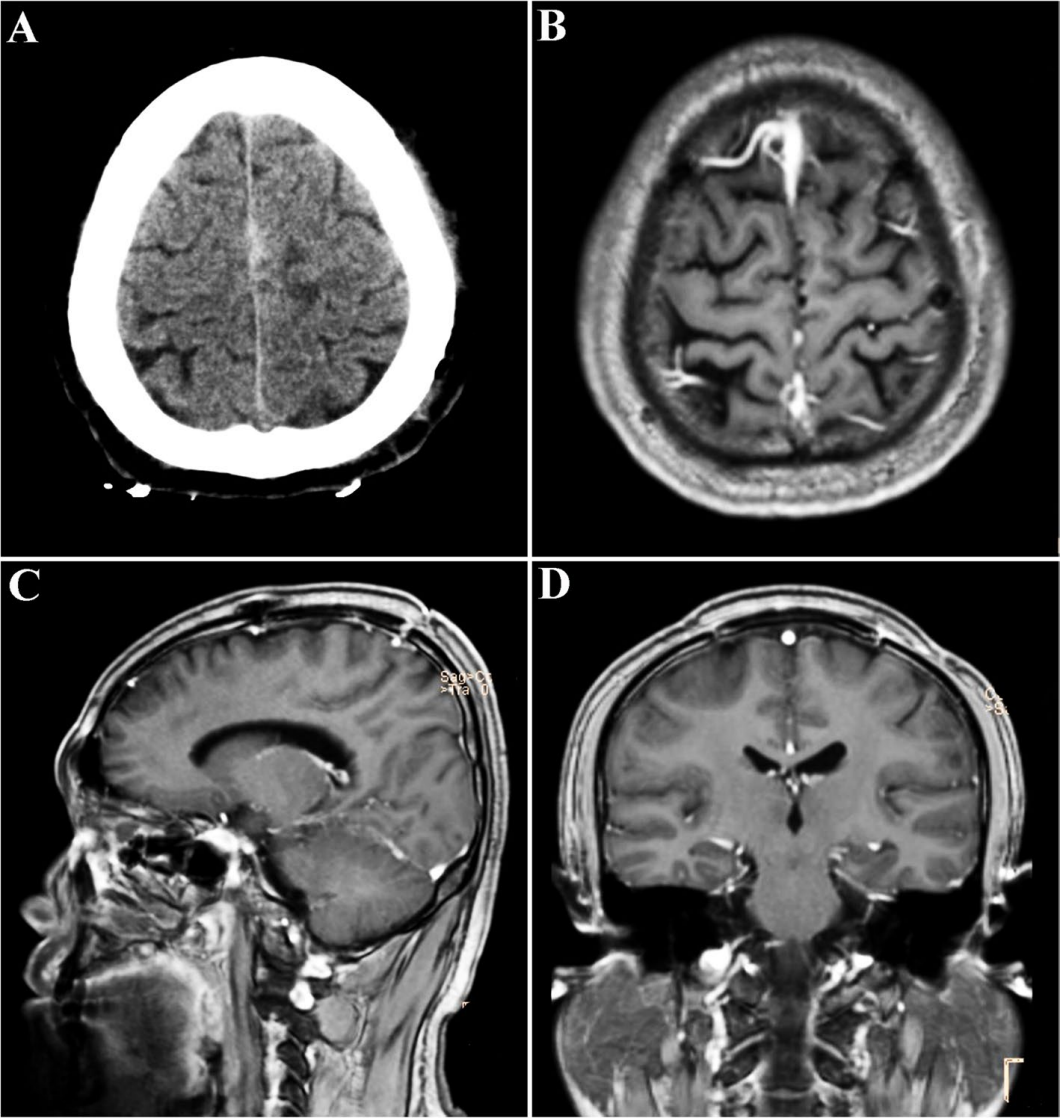
**A** Four hours after operation, the CT showed that the tumor was largely removed, and there was no obvious bleeding in the operation area. Head MRI at 9 months postoperative revealed no tumor recurrence. **B** Axial enhanced MRI 9 months after operation. **C** Sagittal enhanced MRI 9 months after operation. **D** Coronal enhanced MRI 9 months after operation

The patient underwent further chemotherapy and radiotherapy postoperatively. Nine months after operation, no tumor recurrence was found on enhanced MRI (Fig. 3). The patient had no obvious headache, his limb motor function was normal, and there was no seizure after operation.

## Discussion

Angiosarcoma is a malignant tumor originating from vascular endothelial cells(1). Primary meningeal angiosarcoma is very rare. At present, only 8 cases of primary meningeal angiosarcoma have been reported (Table 1). Primary intracranial angiosarcoma is a rare malignant angiogenic tumor, and it is difficult to make accurate diagnosis before surgery [3]. The diagnosis of primary intracranial angiosarcoma depends on the clinical manifestation, radiologic findings and pathological features.

**Table 1:** Cases of primary intracranial meningeal angiosarcoma in the literature

Report	Age	Sex	Location	Therapy	Overall Survival
Mena et al. 1991 [3]	Unknown	Unknown	Unknown	Surgery	> 39 months
Kirk et al., 1992 [4]	2 weeks	Female	Right temporal	Surgery	> 26 months
Antoniadis et al.,1996 [5]	41 years	Female	Left parietal	Surgery + radiotherapy + chemotherapy	> 41 months
Guode et al. 2008 [6]	16 years	Female	Cerebellopontine angle	Surgery + radiotherapy	> 6 months
Hackney et al. 2012 [7]	35 years 47 years	Female Male	Anterior skull base	Surgery + radiotherapy + chemotherapy	> 18 months Unknown
Sakai et al. 2014 [8]	33 years	Male	Cerebellopontine angle	Surgery + radiotherapy	27 months
Melguizo-Gavilanes et al. 2020 [9]	36 years	Male	Left occipital	Surgery + radiotherapy + chemotherapy	18 months
Mhatre et al. 2021 [10]	30 years	Male	Left parietal	Surgery + radiotherapy + chemotherapy	Unknown
This study	48 years	Male	Parasagittal	Surgery + radiotherapy + chemotherapy	> 16 months

The clinical symptoms of primary intracranial angiosarcoma depend on the anatomical site of the tumor and the increased intracranial pressure caused by mass effect [5]. Acute exacerbation of clinical symptoms may be due to rapid tumor growth or intratumor hemorrhage [11]. Radiotherapy, arsenic exposure and history of previous trauma are known risk factors for extracranial angiosarcoma, but there are no clear risk factors for intracranial angiosarcoma [1]. Due to the high recurrence rate, radical surgical resection is recommended [12]. In this case, the lesion presented with unclear boundaries, uneven enhancement, and somewhat similar appearance to hemorrhagic lesions. Although primary extracranial angiosarcoma metastasis to the central nervous system from other sites have been previously reported [13], there was no radiographic or clinical evidence in our patient to support metastasis to the central nervous system from other sites.

Intraoperatively, there was extensive hemosiderin deposition on the cortical surfaces of the frontal and parietal lobes around the tumor, possibly due to persistent mild bleeding in the lesion. This finding suggests a vasogenic tumor. Superficial hemosiderosis of the central nervous system is a rare pathological condition characterized by deposition of hemosiderin on the pia mater and the surface of brain tissue following chronic (usually asymptomatic) hemorrhage in the subarachnoid space [6, 11]. This unusual intraoperative feature may be characteristic of intracranial angiosarcoma.

The histopathological feature of this case is angiosarcoma, with pleomorphic spindle cells and epithelial cells organizing into abnormal new vessels. In poorly differentiated tumors, endothelial lineages must be determined by immunohistochemistry [14]. angiosarcoma has characteristic expression of endothelial markers CD31, CD34, factor VIII associated antigens and vascular endothelial growth factor [15, 16]. The vascular marker CD31 has relative specificity and good sensitivity and is positive in approximately 90% of primary soft tissue angiosarcomas [14]. CD34 is a useful but less specific marker that is positive in many other tumors, including solitary fibromas and some meningiomas [2].

The imaging appearance of intracranial angiosarcoma depends on the extent of intratumor hemorrhage [11]. Common imaging features of intracranial angiosarcoma could not be identified, and some cases showed uneven signal intensity on T1—and T2-weighted images with mild enhancement, suggesting the presence of multistage blood degrading components resembling cavernous hemangioma [1]. In addition, some cases showed low signal intensity with scattered patchy hypersignal shadow and uneven enhancement with necrosis on T1-weighted images [9]. Most cases have perifocal edema. Although the incidence of intracranial angiosarcoma is extremely low, the clinician should consider primary or metastatic intracranial angiosarcoma when radiologic examination reveals rapid growth and extensive hemorrhagic lesions with perifocal edema [11].

The tumor in this case is closely related to the meninges and is easily misdiagnosed as haemangiopericytoma before surgery. The CT scan of haemangiopericytoma usually shows slightly higher density and no calcification within the lesion. The MRI imaging of haemangiopericytoma is complex, with equal or low signal intensity on T1WI and mixed signal intensity on T2WI. The tumor area of meningeal hemangiopericytoma has abundant blood supply, and some can see vascular flow void signals; The enhanced MRI scan showed significant uneven enhancement of the tumor, with no significant enhancement of necrotic cystic lesions within it [17]. In addition, intracranial angiosarcoma is often confused meningiomas, cavernous hemangiomas, hemorrhagic metastases, and high-grade gliomas. Immunohistochemistry is very important in the differential diagnosis of intracranial angiosarcoma.Some immunohistochemical markers such as ERG, vementin, S100, HMB-45, MIB-1, UEA-1, and GFAP, are particularly useful in making differential diagnosis with hemangioblastoma, Kaposi’s sarcoma, hemangioendothelioma, cavernous angioma, carcinoma metastases, and epithelious melanoma [3, 9].

Regarding the treatment of intracranial angiosarcoma, all the previously published literatures are retrospective case reports without evidence based on clinical trials. Total resection is generally recommended for primary and metastatic CNS angiosarcomas [5, 18]. Adjuvant or stereotactic radiotherapy is commonly used to improve local control rates [5]. Radiotherapy has been shown to be beneficial in improving disease controland has been used as an adjunct to surgical treatment or as palliative treatment. Scholsem et al. and Khan et al. reported the use of adjuvant radiotherapy at the tumor site with good local control [15, 18].

In addition, chemotherapy is a common treatment. However, the effectiveness of chemotherapy is generally poor, which makes chemotherapy only play a role of palliative treatment [7]. Paclitaxel, adriamycin, taxane, gemcitabine, temozolomide, thalidomide, sorafenib, sunitinib and bevacizumab have been used to treat angiosarcoma [16, 18], but response rates are generally poor. The current postoperative chemoradiotherapy regimen is: postoperative radiotherapy of 60 Gy, combined with temozolomide 75 mg/(m^2^·d) and bevacizumab (10 mg/kg) for 2 weeks, and given temozolomide and bevacizumab for maintenance treatment [2]. Traditional drugs such as paclitaxel, adriamycin and gemcitabine do not penetrate the central nervous system effectively, so the role of palliative chemotherapy needs further clinical validation.

Prognosis of intracranial angiosarcoma varies greatly among individuals, with some reports of long-term survivals [3]. Characteristics associated with poor prognosis include tumor site, size, and resectability [2]. The prognosis of lesions located on the meninges [7, 10] was reported to be superior to that of intracerebral lesions. In our case, the tumor is located in the parasagittal dura mater, and the prognosis of patient after surgery is better than that of patients with tumor located in brain tissue. Differences in individual survival suggest heterogeneity of intracranial angiosarcoma and inaccuracy of current pathological grading. Therefore, it is necessary to establish accurate pathological classification to better predict the prognosis of individual cases.

## Conclusion

We report a rare case of primary intracranial parasagittal meningeal angiosarcoma and describe its clinical presentation, imaging features, and clinical course. Although accurate preoperative diagnosis is difficult, radiologists and neurosurgeons need to be aware of this rare tumor. The presence of extensive superficial hemosiderin deposition during surgery may be helpful in diagnosis, and immunohistochemistry is very important for the diagnosis of the tumor.

## Data Availability

The raw data supporting the conclusions of this article will be made available by the authors, without undue reservation.
